# Children at Risk: The Growing Impact of USAID Cuts on Pediatric Malnutrition and Death Rates

**DOI:** 10.1111/mcn.70028

**Published:** 2025-03-17

**Authors:** Zainab Anfaal, Muneeb Khawar, Javed Iqbal, Shree Rath

**Affiliations:** ^1^ Khyber Medical College Peshawar Pakistan; ^2^ King Edward Medical University Lahore Pakistan; ^3^ Hamad Medical Corporation Doha Qatar; ^4^ All India Institute of Medical Sciences Bhubaneswar India

To the Editor,

The recent withdrawal of USAID funding has intensified a growing humanitarian crisis, disproportionately affecting the world's most vulnerable children under five suffering from malnutrition. For decades, USAID‐funded nutrition programs have been a critical lifeline, providing food aid, maternal health support, and emergency nutrition interventions. With the sudden loss of this support, millions of children now face an increased risk of severe malnutrition, developmental delays, and death from preventable causes. Without urgent intervention, the gains made in child survival and public health over the last two decades will be reversed.

According to the World Health Organization (WHO), malnutrition contributes to 45% of all child deaths worldwide. As of 2022, the numbers are staggering: 149 million children suffer from stunting (low height for age), 45 million experience wasting (low weight for height), and 37 million are overweight, highlighting a growing double burden of malnutrition (Fact sheets—Malnutrition 2025). USAID‐supported programs have historically played a pivotal role in reducing these numbers by funding community‐based management of acute malnutrition (CMAM) programs, ensuring early diagnosis and treatment; providing ready‐to‐use therapeutic foods (RUTF), which offer lifesaving nutrition to children suffering from severe acute malnutrition (SAM); and supporting food fortification initiatives to address micronutrient deficiencies like iron and vitamin A that contribute to higher child mortality rates. The withdrawal of USAID funding threatens to reverse progress in all these areas, particularly in low‐income countries like Pakistan, Yemen, and South Sudan, where childhood malnutrition is already at crisis levels.

The funding cuts have already had an immediate impact, leading to increased child mortality as children suffering from malnutrition become more vulnerable to preventable diseases like diarrhea, pneumonia, and measles (FACTBOX—USAID Cuts: Why Trump's Funding Freeze Threatens Millions Worldwide 2025). The loss of maternal and child nutrition programs will also result in higher rates of stunting and wasting, leading to lifelong health and cognitive impairments that reduce educational and economic opportunities (Savage 2025). Furthermore, the worsening food insecurity means that many families, who rely on USAID‐funded nutrition programs to access affordable and fortified food, will face greater challenges in meeting their children's nutritional needs. Experts are warning that without alternative funding sources, we may witness a catastrophic rise in malnutrition‐related deaths in the coming years.

The crisis demands immediate intervention from governments, global health agencies, and private stakeholders. Emergency international funding is critical to replace USAID support. Organizations like UNICEF, WHO, and the World Bank must coordinate efforts to sustain malnutrition treatment and prevention programs. Governments in high‐risk countries should also increase domestic investments in child nutrition programs, while public–private partnerships should be leveraged to ensure food security and nutritional support. At the 2021 Nutrition for Growth Summit, USAID Administrator Samantha Power highlighted the importance of US investments, announcing plans to allocate up to $11 billion over 3 years to combat global malnutrition—the underlying cause of almost half of childhood deaths globally (U.S. Agency for International Development [USAID] 2025).

Strengthening community‐based nutrition programs is another essential step. Expanding nutrition education initiatives on breastfeeding, complementary feeding, and hygiene will help prevent malnutrition‐related infections. Scaling up CMAM programs will enable earlier identification and treatment of children at risk. Moreover, timely distribution of RUTF and fortified foods must be prioritized in high‐burden regions to prevent further escalation.

Finally, policy interventions and advocacy are necessary. Global leaders must advocate for the restoration of USAID funding for malnutrition programs, and national governments should prioritize food fortification and school feeding programs. Long‐term solutions such as improving food systems and maternal health care should also be part of the agenda, aiming to reduce reliance on external aid in the future. Experts have voiced urgent concerns about the impact of the USAID funding freeze on global health programs, calling for immediate intervention from governments, international agencies, and private stakeholders to sustain these vital initiatives (Medical Experts Concerned About USAID Spending Cuts Could Impact Global Health Programs 2025).

The loss of USAID funding for malnutrition programs is a direct threat to the health and survival of millions of children worldwide. Without immediate intervention, the progress made in reducing child mortality and malnutrition will be undone. The global community must act swiftly to bridge the funding gap, implement sustainable solutions, and prevent a worsening humanitarian disaster. The lives of millions of children depend on it.
